# R152C DNA Pol β mutation impairs base excision repair and induces cellular transformation

**DOI:** 10.18632/oncotarget.6849

**Published:** 2016-01-08

**Authors:** Ting Zhou, Feiyan Pan, Yan Cao, Ying Han, Jing Zhao, Hongfang Sun, Xiaolong Zhou, Xuping Wu, Lingfeng He, Zhigang Hu, Haoyan Chen, Binghui Shen, Zhigang Guo

**Affiliations:** ^1^ Jiangsu Key Laboratory for Molecular and Medical Biotechnology, College of Life Sciences, Nanjing Normal University, Nanjing, China 210023; ^2^ Division of Gastroenterology and Hepatology, RenJi Hospital, School of Medicine, Shanghai Jiao Tong University, Shanghai, China 200001; ^3^ The Second Hospital of Nanjing, the Second Affiliated Hospital of Southeast University, Nanjing, China 210003; ^4^ Department of Cancer Genetics and Epigenetics, City of Hope National Medical Center and Beckman Research Institute, Duarte, CA, USA 91010

**Keywords:** DNA polymerase β, BER, DNA damage, tumorigenesis, genome stability

## Abstract

DNA polymerase β (Pol β) is a key enzyme in DNA base excision repair (BER), a pathway that maintains genome integrity and stability. Pol β mutations have been detected in various types of cancers, suggesting a possible linkage between Pol β mutations and cancer. However, it is not clear whether and how Pol β mutations cause cancer onset and progression. In the current work, we show that a substitution mutation, R152C, impairs Pol β polymerase activity and BER efficiency. Cells harboring Pol β R152C are sensitive to the DNA damaging agents methyl methanesulfonate (MMS) and H_2_O_2_. Moreover, the mutant cells display a high frequency of chromatid breakages and aneuploidy and also form foci. Taken together, our data indicate that Pol β R152C can drive cellular transformation.

## INTRODUCTION

Genomic DNA is constantly exposed to endogenous and exogenous insults, which cause DNA damage. If not repaired, this DNA damage may result in genetic mutations, leading to genome instability and cancer initiation [1, 2]. Removal of DNA damage and maintenance of genomic integrity depend on robust cellular DNA repair systems [2]. Base excision repair (BER), which removes DNA base damage caused by endogenous and exogenous agents, is a major repair pathway in eukaryotic cells [3–6]. It is estimated that BER repairs about 10^4^ damaged/modified bases per cell per day [7–9]. BER is initiated with the excision of the damaged base by a specific DNA glycosylase, resulting in an apurinic/apyrimidinic site (AP site). The AP site is then cleaved by AP endonuclease 1 (APE1), leaving 3′ hydroxyl and 5′ deoxyribosephosphate termini (5′-dRp) [10, 11]. This intermediate structure can be processed through either the short patch BER (SP-BER) or the long patch BER (LP-BER) pathway [12, 13]. In SP-BER, Pol β adds only one nucleotide to the 3′-end of the nicked AP site, and then the dRP lyase activity of Pol β catalyzes β-elimination of the 5′-sugar phosphate residue, resulting in a ligatable nick that can then be sealed by XRCC1/Ligase IIIα [14, 15]. In LP-BER, Pol β performs strand displacement synthesis, generating a 2-10 nt short DNA flap, which is removed by flap endonuclease 1 (FEN1) [16–20]. DNA ligase I then seals the nick [12].

DNA polymerase β (Pol β) is a key player in both the SP-BER and LP-BER pathways [19, 21, 22]. Pol β, a 39 kDa protein, contains two domains, a dRP lyase domain (8 kDa) and a polymerase domain (31 kDa). These two domains correspond to the dRP lyase and polymerase activities, which are responsible for the removal of the sugar phosphate group and the incorporation of new deoxyribonucleotides, respectively [23]. The Pol β polymerase domain can be divided into the fingers, palm and thumb subdomains, based on crystallographic structures. These subdomains are responsible for dsDNA binding, nucleotidyl transferase and dNTP selection, respectively [23–25]. In addition, Pol β also interacts with many other proteins including APE1, PCNA and FEN1 [26, 27]. These interactions can recruit downstream factors to the DNA repair site, reciprocally stimulate enzyme activities, and coordinate the highly ordered chemical reactions of BER. Pol β deficiency impairs BER efficiency and promotes hypersensitivity to alkylating or oxidative agents [18, 28]. Knockout of Pol β in mice abolishes BER and induces hypersensitivity to DNA damaging reagents, including methyl methanesulfonate (MMS) and H_2_O_2_, leading to early embryonic lethality.

**Figure 1:**
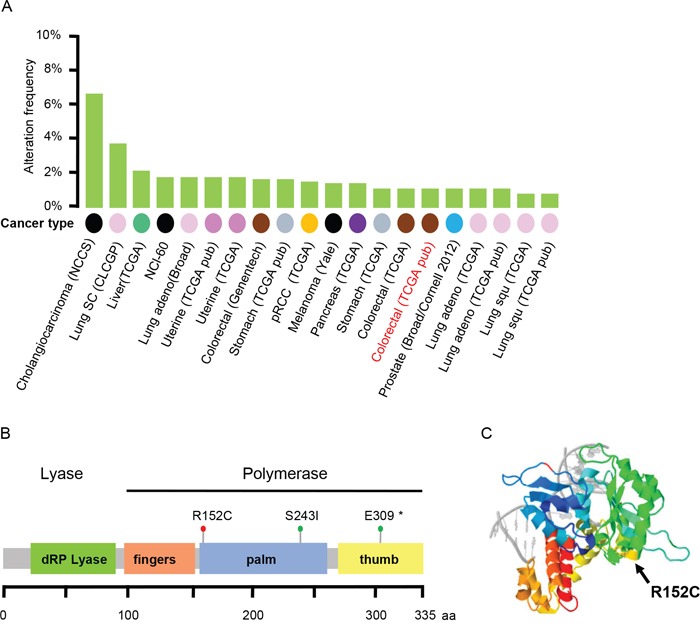
The R152C mutation of Pol β in colorectal cancer **A.** Pol β mutation frequency across each cancer type in the Cancer Genome Atlas (TCGA) dataset. **B.** Three colorectal cancer-associated Pol β mutations from the TCGA dataset. **C.** The position of R152C in the 3D structure of Pol β.

Most types of human cancers contain Pol β mutations (Figure 1A, [29, 30]). These findings suggest that Pol β mutation may promote carcinogenesis [31–35], but a clear link between Pol β mutation and cancer has not been established. In the current study, we identified Pol β R152C as a candidate cancer-causing mutation. We expressed and purified Pol β WT and R152C and found that R152C significantly reduces Pol β polymerase activity and BER efficiency. Furthermore, cells harboring Pol β R152C accumulated more genomic DNA damage in response to DNA damaging agents, which induced aneuploidy and a higher cellular transformation efficiency. These results suggest that the R152C mutation reduces Pol β biochemical activity, resulting in defective BER, which might contribute to genome instability and cancer development.

## RESULTS

### The R152C mutation of Pol β is associated with colorectal cancer

By searching the Cancer Genome Atlas (TCGA) Data, we found that the Pol β mutation exists in all human tumors (Figure 1A). In colorectal cancer samples, we found 3 Pol β mutations: R152C, S243I, and E309* (*stop codon) (Figure 1B). Among the three mutations, R152C, an amino acid substitution of arginine by cysteine, was interesting because of its location on the protein, its effect on the net charge of the protein, and the sequence conservation of R152 among different species (Figure 1C) [36]. Furthermore, R152 has also been reported to be a methylation site of DNA Pol β [37]. Methylation on R152 could enhance the Pol β DNA binding activity and stimulate its DNA polymerase activity. The R152C mutation is methylation defective and, therefore, we speculated that this mutation could disrupt cellular functions.

### The R152C mutation is defective in polymerase activity

**Figure 2:**
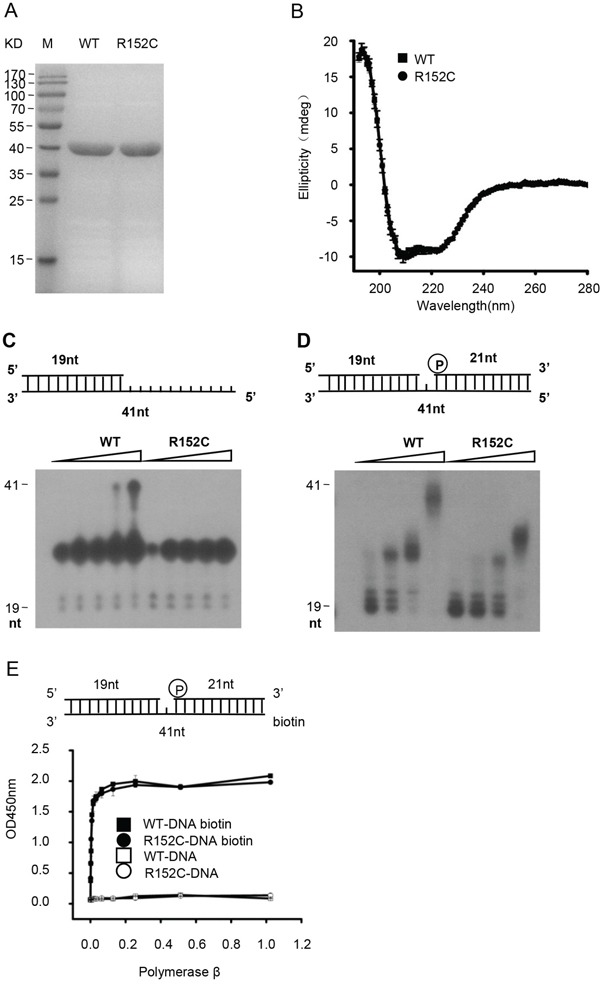
The Pol β R152C mutant is defective in polymerase activity **A.** SDS-PAGE of the Pol β WT and R152C recombinant protein. Proteins were expressed in *E. coli* and purified by a His-tag column. **B.** Circular dichroism spectroscopy analyses of WT and R152C Pol β. **C.** Pol β primer extension activity assay. **D.** Pol β gap-filling activity assay. In (C) and (D), the top part of each panel shows the schematic structure of the corresponding DNA substrates. The bottom shows PAGE-separated products. **E.** ELISA-based isotherm adsorption assay of the DNA-binding affinity of WT and R152C Pol β.

We first determined the effects of R152C mutation on Pol β function. WT and R152C Pol β were purified from *E. coli* to homogeneity (Figure 2A). Circular dichroism analysis showed that WT and R152C Pol β had the same overall structure (Figure 2B). We then assayed primer extension, gap filling, dRP lyase, and substrate-binding activity *in vitro*, using synthetic DNA substrates (Table 1). We found that R152C mutation dramatically reduced primer extension (Figure 2C) and gap filling (Figure 2D) activity compared to the WT enzyme. However, no differences were observed for DNA-binding activity (Figure 2E) or dRP lyase activity (data not shown). These results were consistent with the fact that R152C is located in the 31 kD polymerase catalytic domain, whereas the 8 kD domain is responsible for dRP lyase and DNA-binding activity.

**Table 1:** Prime and DNA substrates

Name	Oligonucleotide sequence	Application
Pol β R152C F	5′-gggactttgaaaaaagaattccttgtgaagagatgttacaaatg-3′	Mutagenesis
Pol β R152C R	5′-ttgcatttgtaacatctcttcacaaggaattcttttttcaaagtccc-3′	
Pol β -2oli	5′-ct tacacgttgact accgt 5′-ggatccactcttgcctcaaaagacggtagtcaacg tgtaag-3′	Polymerase activity
Pol-β -3oli	5′-ct tacacgttgact accgt ttttgaggcaagagt ggat cc-3 5′-ggatccactcttgcctcaaaagacggtagtcaacgtgtaa g-3′	Polymerase activity & DNA binding assay
Pol β -U	5′-c ttacacgttgac tacctt**U**tttgaggcaagagtgga t cc-3′ 5′-ggatc cactc ttgcc tcaaa gaagg tagtcaacgt gtaag-3′	SP-BER assay
Pol β -F	5′-c ttacacgttgac tacctt **F**tttgaggcaagagtgga tcc-3′ 5′-ggatccactcttgcctcaaagaaggtagtcaacgtgtaag-3′	LP-BER assay

### The R152C mutation has lower BER efficiency

**Figure 3:**
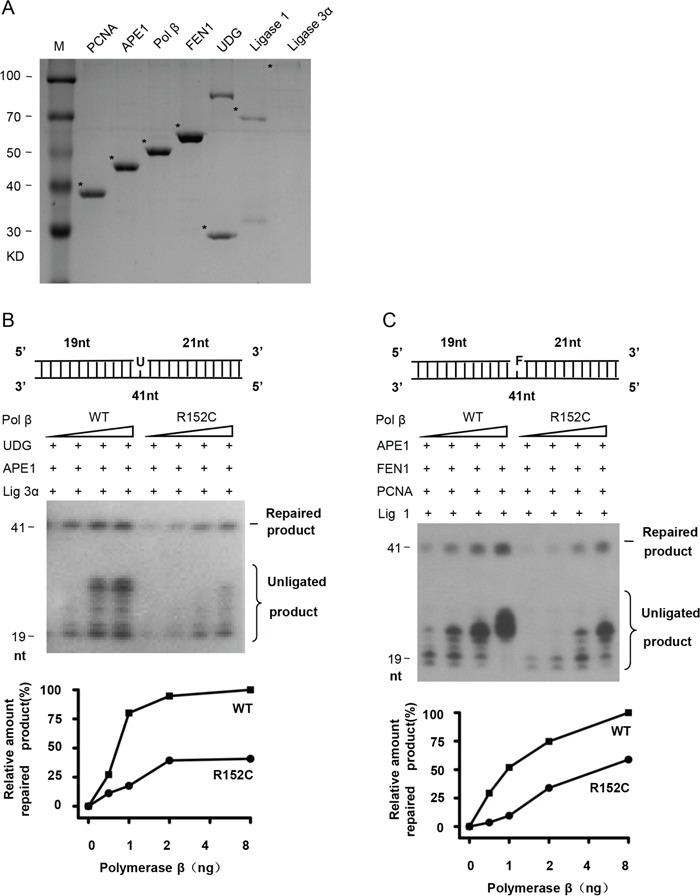
R152C mutation impairs BER efficiency *in vitro* **A.** SDS-PAGE of purified BER proteins. SP-BER **B.** or LP-BER **C.** reconstitution with purified BER proteins. The top part of each panel shows the schematic structures of the corresponding DNA substrates. The middle shows PAGE-separated products and the bottom the relative percentage of repaired product obtained with the indicated amounts of Pol β. Values represent the mean ±SD of three independent assays.

Because Pol β is a key enzyme for BER, we speculate that R152C mutation of Pol β, which impairs its polymerase activity, may also impair its BER function. To test this, BER proteins were purified (Figure 3A). SP- and LP-BER were assayed using G/U mismatched oligonucleotides to mimic the SP-BER substrate and G/F (tetrahydrofuran, THF or F) mismatched oligonucleotides to mimic the LP-BER substrate (Pol β-F) (Table 1). As shown in Figure 3B and 3C, R152C has significantly lower SP- and LP-BER efficacy.

**Figure 4:**
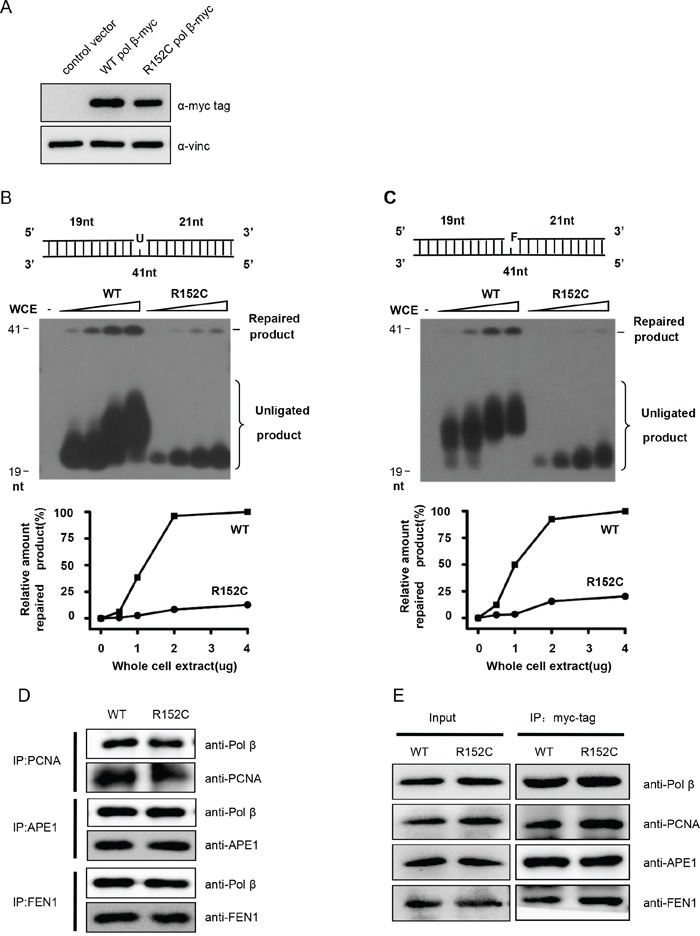
R152C mutation impairs BER efficiency *in vivo* **A.** Western blotting shows expression of exogenous Pol β in 293 cells. SP-BER **B.** or LP-BER **C.** reconstitution with whole cell extracts (WCEs). The top part of each panel shows the schematic structures of the corresponding DNA substrates. The middle shows PAGE-separated products and the bottom the relative percentage of repaired product obtained with the indicated amounts of Pol β. The values represent the mean ±SD of three independent assays. **D.** Pull-down assay. Purified PCNA, APE1 and FEN1 were mixed in Tris buffer. The interaction between two proteins was determined by pull-down assay and western blotting. **E.** Co-immunoprecipitation assay. C-myc-tagged WT or R152C Pol β was expressed in 293 cells. C-myc-tagged proteins were precipitated using an anti-c-myc antibody, followed by western blot analysis using PCNA, APE1 and FEN1 antibodies.

To validate that Pol β R152C reduces cellular BER efficiency, we expressed human WT and R152C Pol β in 293 cells. Cells expressing similar levels of Pol β were selected (Figure 4A). Nuclear extracts (NE) from these cell lines were prepared and their BER efficiencies were assayed. We found that WT NE efficiently repaired the uracil (U) or THF (F) lesion, resulting in a 40 nt band, whereas the repair efficiency by R152C NE was only approximately 10% and 5% for SP-BER and LP-BER, respectively (Figure 4B and 4C).

Pol β has been reported to interact with many other proteins, including APE1 [38–42], PCNA [43], and FEN1 [27, 44]. These interactions play important roles in recruiting downstream factors to the DNA repair site, reciprocally stimulating enzyme activities and coordinating the highly ordered chemical reactions of BER. Disruption of these interactions would impede BER efficiency [27, 45]. To test if R152C disrupts the interaction of DNA Pol β with these proteins, we performed interaction assays *in vitro* (Figure 4D) and *in vivo* (Figure 4E). We found that R152C mutation has no influence on the interaction of Pol β with other major BER proteins, including APE1, FEN1 and PCNA (Figure 4D and 4E), so it is unlikely that defective protein interaction causes the decreased BER efficiency.

### R152C Pol β-expressing cells are hypersensitive to DNA damage

**Figure 5:**
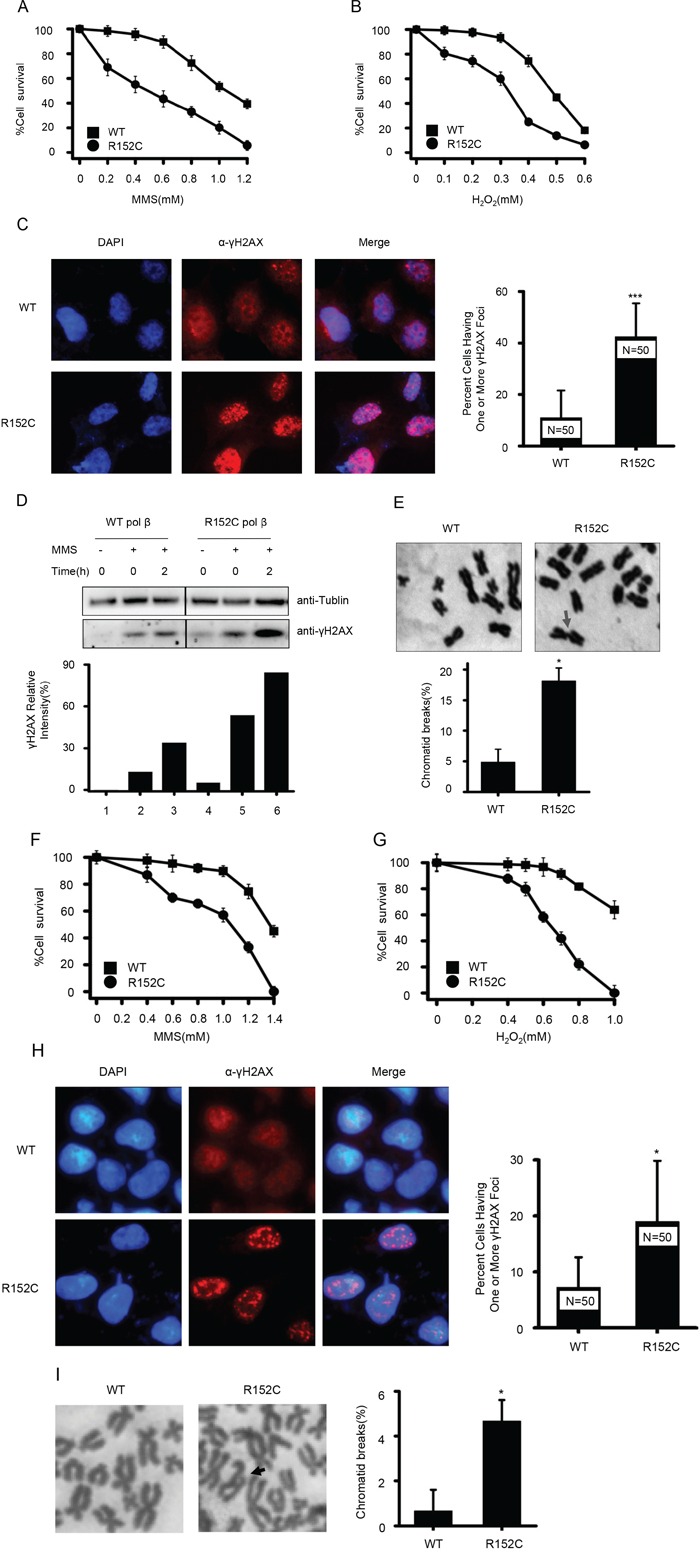
R152C cells are sensitive to the DNA damage agents, MMS and H_2_O_2_ MMS **A.** and H_2_O_2_ **B.** sensitivity assays. WT and R152C 293 cells were treated with MMS (A) and H_2_O_2_ (B) at the indicated concentrations. The number of cells was determined by the CellTiter 96 AQueous one-solution assay. **C.** Immunofluorescence staining for γH2AX (red) in 293 cells. The right panel shows the percentage of cells having one or more γH2AX focus. ***P<0.001, Student's *t*-test. **D.** Western blotting of γH2AX levels before and after MMS treatment. **E.** Giemsa-stained WT and R152C 293 metaphase cells. Arrows indicate chromatid breaks. The bottom panel shows the quantification of mitotic cells with broken chromatids. *P=0.024, Student's *t*-test. MMS **F.** and H_2_O_2_ **G.** sensitivity assays. WT and R152C SW480 cells were treated with MMS (F) and H_2_O_2_ (G) at the indicated concentrations. The number of viable cells was determined by the CellTiter 96 Aqueous one-solution assay. **H.** Immunofluorescence staining for γH2AX (red) in SW480 cells. The right panel shows the percentage of cells possessing one or more γH2AX focus. *P<0.05, Student's *t*-test. **I.** Giemsa-stained WT and R152C SW480 metaphase cells. Arrows indicate chromatid breaks. The right panel shows quantification of mitotic cells with broken chromatids. *P<0.05, Student's *t*-test.

Our data suggested R152C Pol β is defective in BER activity; we therefore suspected that R152C Pol β would sensitize cells to DNA damaging agents such as MMS and H_2_O_2_. This hypersensitivity could lead to accumulation of incompletely repaired DNA intermediates and chromatid breakage. Indeed, R152C Pol β-expressing cells were more sensitive to 1 hour of MMS or H_2_O_2_ treatment, compared with cells harboring WT Pol β (Figure 5A and 5B). To determine whether the R152C Pol β mutation increased the number of double strand breaks (DSBs) in cells, we counted the numbers of γH2AX foci in cells treated with 1 mM MMS. As shown in Figure 5C, more γH2AX foci were found in R152C-expressing cells, which means there were more DSBs. Consistently, western blotting data showed that the cells expressing R152C Pol β mutation increased γH2AX levels, with or without MMS treatment. (Figure 5D).

Because the Pol β R152C mutation was identified from colon cancer cells, we also evaluated the cellular consequences of overexpressing WT or R152C Pol β in SW480 colon cancer cells. We found that SW480 cells harboring R152C Pol β displayed a reduced survival rate (Figure 5F, G) and accumulated more DSBs (Figure 5H) when challenged with DNA damaging agents.

### Pol β R152C cells spontaneously accumulate chromosomal breaks and become aneuploid

Chromosomal instability and aneuploidy are associated with the accumulation of DNA breaks and cancer [46]. Therefore, we compared the number of spontaneous chromosomal breaks that occurred in WT and R152C cells. We found that the 293 cells carrying the R152C Pol β mutation had significantly more chromatid breaks than the WT cells (Figure 5E). This phenomenon was also confirmed in SW480 cells carrying the R152C mutation (Figure 5I).

**Figure 6:**
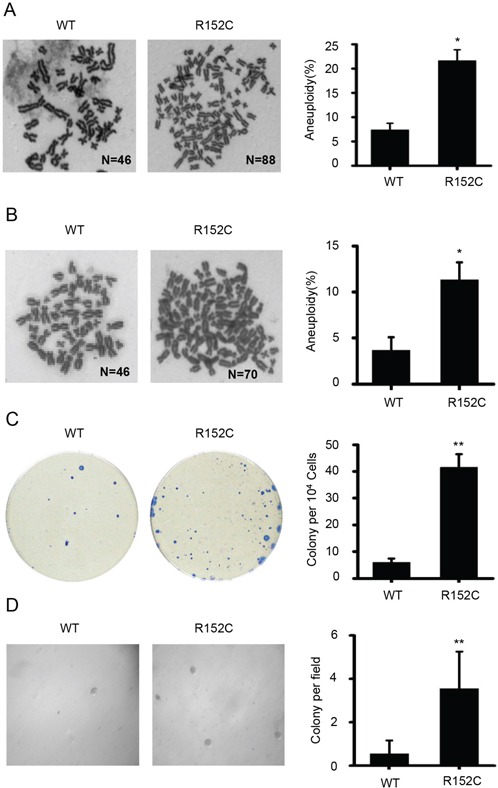
R152C cells have a high transformation potential **A.** WT and R152C 293 metaphase cells were stained by Giemsa. The bottom panel shows the quantification of mitotic cells with aneuploidy, where the number of chromatids was >70. Here, approximately 180 mitotic cells per cell line were analyzed in three independent experiments. *P=0.017, Student's *t*-test. **B.** WT and R152C SW480 metaphase cells were stained by Giemsa. **C.** Image of transformed cell colonies. WT and R152C SW480 cells were plated in a new dish for the colony-focus formation assays. The right panel shows the quantification of colonies, **P<0.01, Student's *t*-test. **D.** Anchorage-independent growth. WT and R152C SW480 cells were plated in a new 6-well dish for anchorage-independent growth. The right panel shows the quantification of colonies, **P<0.01, Student's *t*-test.

The accumulation of chromosomal breaks is associated with aneuploidy. We found that the average percentage of aneuploidy in R152C cells was significantly higher than in WT cells (Figure 6A and 6B). Taken together, the data indicate that Pol β cells have higher levels of spontaneous chromatid breaks and aneuploidy than WT cells.

### R152C cells have a high transformation potential

Our data indicate that R152C cells develop more chromatid breaks and are more likely to become aneuploid. We suspected that these cellular abnormalities would contribute to cellular transformation and lead to clonal expansion. To determine whether the R152C mutation promoted tumorigenesis, we performed cellular focus formation assays (Figure 6C). The number of colonies formed by R152C cells was six-fold higher than the number formed by WT cells, suggesting that R152C cells can develop aneuploidy-associated cancer. Anchorage-independent growth assays also showed that R152C Pol β-expressing cells possessed higher transformation activity than WT cells (Figure 6D).

## DISCUSSION

The Pol β R152C variant was recently identified in a colorectal carcinoma, but its role in cancer initiation was unknown. In this manuscript, we show that expression of the R152C colorectal carcinoma-associated Pol β variant in immortalized 293 cells results in cellular transformation. The mechanism of cellular transformation is likely to be genomic instability resulting from impaired BER efficiency by Pol β R152C mutation. This impaired BER activity, which increases the frequency of chromosomal aberrations, has been associated with several different types of cancers.

To further study the effects of the R152C Pol β variant in BER *in vivo*, we transfected human WT and R152C Pol β gene into 293 cells. Because R152C Pol β shows intact DNA binding activity (Figure 2E), we speculate that R152C Pol β competes with WT Pol β for DNA substrate binding, and therefore perturbs WT Pol β activity in cells. Indeed, R152C Pol β expression reduced both the SP-BER and LP-BER efficiency dramatically.

BER failure would lead to the accumulation of DNA damage, chromosome breakage, and aneuploidy [47]. In our study, R152C Pol β variants displayed defects in SP- and LP-BER. Furthermore, R152C Pol β expression, in both the 293 and SW480 cell lines, increased the sensitivity to MMS treatment, and increased the number of DSBs. In addition, karyotype analysis of R152C Pol β-expressing cells revealed more chromatid breaks and more aneuploid cells than WT controls. Cells usually have a precise surveillance system to detect DNA damage and chromatid aberrations, then they decide whether to die or not. However, a few cells with abnormal chromosomes could successfully bypass this surveillance system and transform into tumor cells. Our focus formation assay revealed that R152C cells formed clones, supporting a model where chromosome instability increases transformation and carcinogenesis [48, 49]. Altogether, our data demonstrate that the R152C mutation diminishes Pol β polymerase activity, impairs its capacity to conduct SP- and LP-BER, and promotes chromosome aberrations and genome instability, which contributes to carcinogenesis. Our results also indicate that R152 of Pol β could be a useful target for the diagnosis, prevention and treatment of cancer [11, 50, 51].

## MATERIALS AND METHODS

### Reagents and antibodies

All primers and DNA substrates used in this paper were synthesized by GenScript, Inc. using polyacrylamide gel electrophoresis (PAGE) purification. Four deoxynucleotide triphosphates (dNTPs) were purchased from New England Biolabs (N0446S). [γ-^32^P]-ATP (BLU002A, 250 uCi) and [α-^32^P]-dCTP (NEG513H, 250uCi) were purchased from PerkinElmer. The Pol β-specific antibody (ab26343) was purchased from Abcam. The anti-PCNA (SC-7907), APE1 (SC-55498), FEN1 (SC-56675), c-Myc (SC-40), Tubulin (sc-23950), goat anti-rabbit IgG-HRP (SC-2004), and goat anti-mouse IgG-HRP (sc-2005) antibodies were purchased from Santa Cruz Biotechnology.

### Plasmid construction and preparation of recombinant proteins

Human WT Pol β cDNA (Genbank accession NM_002690) was from Dr. Binghui Shen's laboratory in City of Hope National Medical Center, California, USA. The R152C mutant was generated using the QuikChange site-directed mutagenesis kit using the primers shown in Table 1. All recombinant proteins, including WT and R152C Pol β, FEN1, APE1, and Ligase IIIα, were purified from *E.coli.*

### Circular dichroism spectroscopy assay

CD spectroscopy analysis was performed as previously described [52]. Purified WT and R152C Pol β proteins were diluted to 1 μM with KH_2_PO_4_, and their CD spectra were detected at 20°C using a Chirascan CD spectrometer. Measurements were collected at intervals of 1 nm from 190 to 280 nm.

### DNA polymerase activity assay

DNA substrates (Table 1) were incubated with 20 μl of reaction buffer A (50 mM Tris-HCl [pH 8.0], 10 mM MgCl2, 2 mM DTT, 20 mM NaCl, 10% glycerol), 50 μM of each dATP, dGTP, and dTTP (NEB), 8 μM [α-^32^P]-dCTP, and various amounts (0-20 ng) of WT or R152C recombinant Pol β for 30 mins at 37°C. The reaction was then stopped by adding equal volumes of gel loading buffer (90% formamide dye, 3 M EDTA, 0.02% bromophenol blue and 0.02% xylene cyanol), heated (5min, 95°C), separated by 15% PAGE containing 8 M urea, and visualized by autoradiography.

### Base excision repair assay

The BER assay was performed as described previously [26, 39, 53, 54]. The reactions were carried out in 20 μl of reaction buffer B (40 mM HEPES-KOH [pH 7.8], 70 mM KCI, 7 mM MgCl_2_, 1 mM dithiothreitol, 0.5 mM EDTA, 2 mM ATP, 50 μM each of dATP, dTTP, and dGTP, and 8 μM 2 μCi [α-^32^P]-dCTP). For SP reconstitution with purified proteins, Uracil-DNA Glycosylase (UDG, 8 ng), APE1 (2 ng), Ligase IIIα (20 ng) and various amounts of Pol β (0-5 ng), were mixed and incubated with the SP-BER substrate Pol β-U (Table 1). For LP-BER, the Pol β-F substrate (Table 1) was incubated with a mixture of APE1 (2 ng), Pol β (0-5 ng), FEN1 (2 ng), and Ligase I (20 ng). For cell extract reconstitution, the SP- or LP-BER DNA substrate was incubated with the whole cell extract (WCE, 0-5 μg). Reactions (30 mins, 37°C) were then stopped by adding an equal volume of the gel loading buffer and visualized by autoradiography.

### DNA-binding assay

ELISA-based affinity assays was used to assay WT or R152C Pol β and DNA binding. First, a 96-well ELISA plate was coated with streptavidin (1 μg/well) and incubated overnight (4°C). Then, biotin-labeled Pol-GAP DNA substrate (1 pmol/well) was immobilized onto a 96-well plate (overnight, 4°C) and washed 3 times with PBS, followed by the addition of 0-1 ug WT or R152C Pol β recombinant proteins. Binding of Pol β was detected using a rabbit anti-Pol β antibody (Abcam, ab26343) and goat anti-rabbit secondary antibody-conjugated HRP (SC-2004). The color was developed by adding tetramethylbenzidine (TMB) and stopped by addition of 1 M H_2_SO_4_. The OD450 value was read by a microplate reader.

### Immunofluorescence

The cells were cultured in six-well plates containing acid-treated cover slides and incubated overnight. The cover slides were then washed with PBS, fixed with 4% formaldehyde in PBS for 30 min, and then washed with PBS. Triton X-100 (0.05%) was added for 5 min to permeabilize the cells. The slides were blocked with 2% BSA and then incubated with primary antibody. The slides were washed then incubated with secondary antibody conjugated with FITC, followed by washing with PBS and staining with DAPI. The mounted slides were viewed with a Zeiss Axioscope and the images were captured with a charge-coupled device camera.

### MMS and H_2_O_2_ sensitivity assay

293 cells transfected with WT/R152C Pol β-N2 vector and N2-vector were seeded (1,500 cells/well) into 96-well plates, incubated (overnight, 37°C), treated (1 h, 37°C) with 0-1.5 mM MMS or 0-1 mM H_2_O_2_, washed with PBS, returned to fresh medium (DMEM containing 10% FBS), and incubated under normal growth conditions (37°C, 5% CO_2_, 72 h). The number of viable cells was determined by the CellTiter 96 AQueous one-solution cell proliferation assay (Promega). Each dilution of MMS or H_2_O_2_ included at least four replicates. The data are expressed as the percentage of growth relative to untreated controls.

### Cell transformation assays

The focus formation assays were conducted according to a previously established protocol [55, 56]. Briefly, 1×10^4^ cells were plated in 6 cm dishes and incubated for 30 days at 37°C. The cells were then washed with PBS and fixed with 4% formaldehyde in PBS for 30 mins. Giemsa was used to stain the cells overnight at room temperature. Stained plates were washed and dried prior to scoring the colonies. Anchorage-independent growth was assessed as previously described [56]. Approximately 8×10^3^ cells were mixed with media containing 1% noble agar. This mixture was poured onto a layer of media containing 3% noble agar in a well of a 6-well dish and incubated for 30 days at 37°C. The number of colonies present in each of five microscope fields per well from a total of 6 wells per experiment were counted after 4 weeks of growth.
